# Tetra-μ_3_-methano­lato-tetra­kis­[(2-formyl-6-meth­oxy­phenolato)methano­lnickel(II)]

**DOI:** 10.1107/S1600536810043497

**Published:** 2010-10-31

**Authors:** Kouassi Ayikoe, Ray J. Butcher, Yilma Gultneh

**Affiliations:** a Department of Chemistry, Howard University, 525 College Street NW, Washington, DC 20059, USA

## Abstract

The molecule of the title compound, [Ni_4_(CH_3_O)_4_(C_8_H_7_O_3_)_4_(CH_3_OH)_4_], has *S*_4_ symmetry. Each of the four Ni^II^ atoms occupies every other corner of a cube, with the alternate corners occupied by μ_3_-methano­late bridging groups linking to three Ni^II^ atoms. Each Ni^II^ atom is in an O_6_ octa­hedral coordination environment formed by three O atoms from three μ_3_-methano­late groups, one from methanol, and two others from a bidentate 2-formyl-6-meth­oxy­phenolate ligand. The Ni—O bond distances range from 2.0020 (14) to 2.0938 (14) Å, the *cis* bond angles range from 81.74 (6) to 97.63°, and the *trans* bond angles range from 168.76 (5) to 175.22 (6)°. There are bifurcated hydrogen-bonding inter­actions between the coordinated methanol OH groups and both the phenolic and meth­oxy O atoms of an adjoining 2-formyl-6-meth­oxy­phenolate moiety. In addition, there are weak inter­molecular C—H⋯O inter­actions involving the meth­oxy O atoms.

## Related literature

For literature related to Ni_4_ cubane-type clusters, see; Andrew & Blake (1969 ▶); Barnes & Hatfield (1971 ▶); Bertrand *et al.* (1971 ▶, 1978 ▶); Brezina *et al.* (1998 ▶); Cromie *et al.* (2001 ▶); El Fallah *et al.* (1996 ▶); Gladfelter *et al.* (1981 ▶); Luo *et al.* (2007 ▶); Moragues-Canovas *et al.* (2004 ▶); Mukherjee *et al.* (2003 ▶); Ran *et al.* (2008 ▶); Yang *et al.* (2006 ▶).
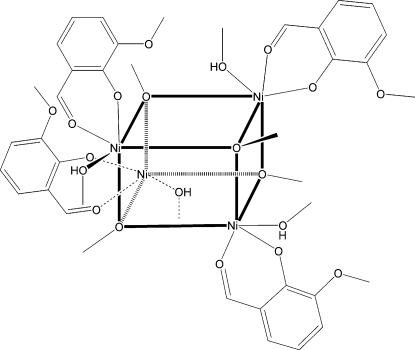


## Experimental

### 

#### Crystal data

[Ni_4_(CH_3_O)_4_(C_8_H_7_O_3_)_4_(CH_4_O)_4_]
*M* *_r_* = 1091.69
Tetragonal, 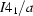

*a* = 22.2670 (9) Å
*c* = 9.70106 (10) Å
*V* = 4810.0 (3) Å^3^
*Z* = 4
Mo *K*α radiation
μ = 1.62 mm^−1^
*T* = 110 K
0.47 × 0.28 × 0.24 mm

#### Data collection

Oxford Xcalibur diffractometer with a Ruby (Gemini Mo) detector
Absorption correction: multi-scan (*CrysAlis PRO*; Oxford Diffraction, 2007 ▶) *T*_min_ = 0.463, *T*_max_ = 1.000
12226 measured reflections
2962 independent reflections
2131 reflections with *I* > 2σ(*I*)
*R*_int_ = 0.035

#### Refinement

*R*[*F* ^2^ > 2σ(*F* ^2^)] = 0.032
*wR*(*F* ^2^) = 0.081
*S* = 0.99
2962 reflections
149 parameters
H-atom parameters constrained
Δρ_max_ = 0.32 e Å^−3^
Δρ_min_ = −0.26 e Å^−3^

### 

Data collection: *CrysAlis PRO* (Oxford Diffraction, 2007 ▶); cell refinement: *CrysAlis PRO*; data reduction: *CrysAlis PRO*; program(s) used to solve structure: *SHELXS97* (Sheldrick, 2008 ▶); program(s) used to refine structure: *SHELXL97* (Sheldrick, 2008 ▶); molecular graphics: *SHELXTL* (Sheldrick, 2008 ▶); software used to prepare material for publication: *SHELXTL*.

## Supplementary Material

Crystal structure: contains datablocks I, global. DOI: 10.1107/S1600536810043497/bt5390sup1.cif

Structure factors: contains datablocks I. DOI: 10.1107/S1600536810043497/bt5390Isup2.hkl

Additional supplementary materials:  crystallographic information; 3D view; checkCIF report

## Figures and Tables

**Table 1:** Hydrogen-bond geometry (Å, °)

*D*—H⋯*A*	*D*—H	H⋯*A*	*D*⋯*A*	*D*—H⋯*A*
O2S—H2S⋯O1^i^	0.84	2.05	2.8062 (19)	150
O2S—H2S⋯O3^i^	0.84	2.50	3.181 (2)	139
C5—H5*A*⋯O3^ii^	0.95	2.45	3.360 (3)	159
C1S—H1S*C*⋯O2S^iii^	0.98	2.47	3.106 (3)	122

Symmetry codes: (i) 
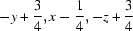
; (ii) 
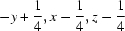
; (iii) 
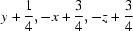
.

**Fig. 1.**
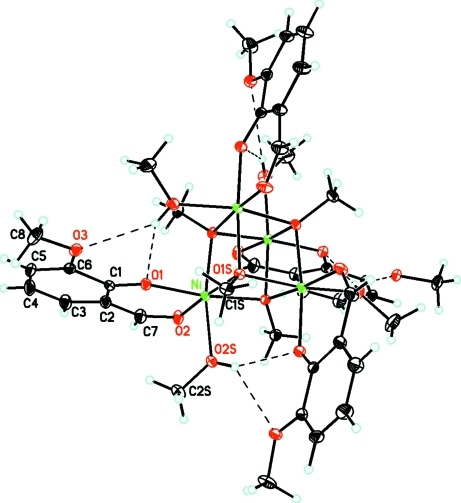
Diagram showing the pseudo-cubic {Ni-(µ3-OCH3)[o-OC6H3(CH3O)CHO](CH3OH)}4 cluster with unique part labelled. The bifurcated intramolecular hydrogen bonds are shown by dashed lines.

**Fig. 2.**
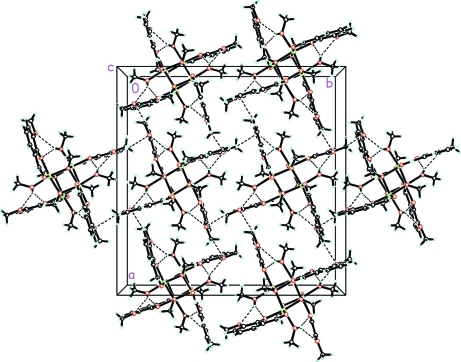
The molecular packing for {Ni-(µ3-OCH3)[o-OC6H3(CH3O)CHO](CH3OH)}4 viewed down the c axis. Intra- and intermolecular interactions are shown by dashed lines.

## supplementary crystallographic information

### Comment

Polynuclear nickel(II) complexes have become a focused research area due to their single-molecule magnet properties, biomimetic activity and their flexibility to engender cluster construction. (Yang *et al.*, 2006). The structural and magnetic properties of symmetric Ni_4_O_4_ cores have been correlated to the Ni—O—Ni angle. It has been shown that the Ni_4_O_4_ core exhibits ferromagnetic interactions when the Ni—O—Ni angle is less than 98° and antiferromagnetic interactions when the angle is greater than 109° (Andrew & Blake, 1969; Barnes & Hatfield, 1971; Bertrand *et al.*, 1971, 1978; Gladfelter *et al.*, 1981; Mukherjee *et al.*, 2003). Moragues-Canovas *et al.* (2004) have synthesized and studied the low-temperature magnetism of the Ni_4_O_4_ cubane core complex with four pendant acetonitrile ions and four nitrate ions. Brezina *et al.* (1998), El Fallah *et al.* (1996), Luo *et al.* (2007), Cromie *et al.* (2001), and Ran *et al.* (2008) have synthesized, crystallized and studied their magnetism at low and/or various temperatures and confirmed the ferromagnetism/antiferromagnetism of such cubane Ni_4_O_4_ core complexes.

In Fig.(1), we report a structure of {Ni-(µ_3_-OCH_3_)[*o*-OC_6_H_3_(CH_3_O)CHO](CH_3_OH)}_4_ which has a Ni_4_O_4_ cubane-type core centre formed from four µ_3_-methanolate O atoms and four nickels. Each Ni^II^ is in an octahedral O_6_ coordination environment completed with three µ_3_-methanolate O atoms, a bidentate 2-formyl-6-methoxyphenolate ligand, and a coordinated methanol molecule. The Ni—O(cubane) bond distances range from 2.0020 (14) to 2.0938 (14) Å, the *cis* bond angles range from 81.74 (6) to 97.63°, and the *trans* bond angles range from 168.76 (5) to 175.22 (6)°. The three Ni—O µ_3_-methanolate bond distances are 2.0350 (13), 2.0568 (13), and 2.0636 (13) Å. All Ni—O distances are within the normal ranges observed in other Ni complexes containing similar ligands. The *o*-vanillin, the methanol, and methanoate cause less distortion about the Ni's due to rigidity and stability established by the cubane Ni_4_O_4_. As a result, this coordination environment of the Ni is closer to perfect octahedral with the following bond angles: O(1*S*)#1-Ni—O(1*S*)#2 81.74 (6)°, O(1*S*)#2-Ni—O(1*S*) 82.32 (5)°, O(1*S*)#1-Ni—O(1*S*) 82.85 (5)°, Ni#1-O(1*S*)—Ni 96.50 (5)°, Ni#2-O(1*S*)—Ni 97.19 (5)°, Ni#2-O(1*S*)—Ni#1 97.91 (6)°. All the bond angles on the cubane (Ni—O—Ni and O—Ni—O) are close to but less than 98°. There are bifurcated hydrogen-bonding interactions between the coordinated methanol OH and both the phenolic and methoxy O of an adjoining 2-formyl-6-methoxyphenolate moiety. In addition there are weak intermolecular C—H···O interactions involving the methoxy O.

### Experimental

The complex was synthesized by reacting 0.53 g (1.45 mmol) of nickel perchlorate [Ni^II^(ClO_4_)_2_.6H_2_O] in methanol [MeOH] (20 ml) with a mixture of 0.23 g *o*-vanillin (1.46 mmol) and 0.26 g of 2-benzylaminopyridine (2-BAP) (1.46 mmol). The secondary amine and the aldehyde were initially mixed in 30 ml of methanol and refluxed with stirring overnight between 50 C and 60 C. The nickel salt solution and the ligands were then mixed and stirred overnight at room temperature (23°-25°), followed by reduced pressure (vacuum) evaporated to obtain a green oily (semi-solid). A portion of the washed product (about 0.025 g) was dissolved in 50/50 MeOH/Propanol. This solution obtained was filtered and layered with diethyl ether. Greenish X-ray quality crystals were obtained after five days of slow diffusion of the diethyl ether into the MeOH/Propanol solvent.

### Refinement

H atoms were placed in geometrically idealized positions and constrained to ride on their parent atoms with a C—H distance of 0.95 *U*_iso_(H) = 1.2*U*_eq_(C) and 0.98 Å for CH_3_ [*U*_iso_(H) = 1.5*U*_eq_(C)]. The H atoms attached to O were idealized with an O—H distance of 0.84 Å.

### Crystal data

**Table:**

[Ni_4_(CH_3_O)_4_(C_8_H_7_O_3_)_4_(CH_4_O)_4_]	*D*_x_ = 1.508 Mg m^−^^3^
*M**_r_* = 1091.69	Mo *K*α radiation, λ = 0.71073 Å
Tetragonal, *I*4_1_/*a*	Cell parameters from 4573 reflections
Hall symbol: -I 4ad	θ = 5.0–29.3°
*a* = 22.2670 (9) Å	µ = 1.62 mm^−^^1^
*c* = 9.70106 (10) Å	*T* = 110 K
*V* = 4810.0 (3) Å^3^	Prism, green
*Z* = 4	0.47 × 0.28 × 0.24 mm
*F*(000) = 2272	

### Data collection

**Table:**

Oxford Xcalibur diffractometer with a Ruby (Gemini Mo) detector	2962 independent reflections
Radiation source: Enhance (Mo) X-ray Source	2131 reflections with *I* > 2σ(*I*)
graphite	*R*_int_ = 0.035
Detector resolution: 10.5081 pixels mm^-1^	θ_max_ = 29.4°, θ_min_ = 4.9°
ω scans	*h* = −21→30
Absorption correction: multi-scan (*CrysAlis PRO*; Oxford Diffraction, 2007)	*k* = −30→22
*T*_min_ = 0.463, *T*_max_ = 1.000	*l* = −13→12
12226 measured reflections	

### Refinement

**Table:**

Refinement on *F*^2^	Primary atom site location: structure-invariant direct methods
Least-squares matrix: full	Secondary atom site location: difference Fourier map
*R*[*F*^2^ > 2σ(*F*^2^)] = 0.032	Hydrogen site location: inferred from neighbouring sites
*wR*(*F*^2^) = 0.081	H-atom parameters constrained
*S* = 0.99	*w* = 1/[σ^2^(*F*_o_^2^) + (0.0454*P*)^2^] where *P* = (*F*_o_^2^ + 2*F*_c_^2^)/3
2962 reflections	(Δ/σ)_max_ = 0.001
149 parameters	Δρ_max_ = 0.32 e Å^−^^3^
0 restraints	Δρ_min_ = −0.26 e Å^−^^3^

### Special details

**Table:**

Geometry. All e.s.d.'s (except the e.s.d. in the dihedral angle between two l.s. planes) are estimated using the full covariance matrix. The cell e.s.d.'s are taken into account individually in the estimation of e.s.d.'s in distances, angles and torsion angles; correlations between e.s.d.'s in cell parameters are only used when they are defined by crystal symmetry. An approximate (isotropic) treatment of cell e.s.d.'s is used for estimating e.s.d.'s involving l.s. planes.
Refinement. Refinement of *F*^2^ against ALL reflections. The weighted *R*-factor *wR* and goodness of fit *S* are based on *F*^2^, conventional *R*-factors *R* are based on *F*, with *F* set to zero for negative *F*^2^. The threshold expression of *F*^2^ > σ(*F*^2^) is used only for calculating *R*-factors(gt) *etc*. and is not relevant to the choice of reflections for refinement. *R*-factors based on *F*^2^ are statistically about twice as large as those based on *F*, and *R*-factors based on ALL data will be even larger.

### Fractional atomic coordinates and isotropic or equivalent isotropic displacement parameters (Å^2^)

**Table:**

	*x*	*y*	*z*	*U*_iso_*/*U*_eq_	
Ni	0.478914 (11)	0.183990 (11)	0.26340 (2)	0.02258 (10)	
O1	0.39721 (6)	0.14647 (6)	0.26250 (13)	0.0269 (3)	
O2	0.48379 (6)	0.17818 (7)	0.05257 (15)	0.0344 (4)	
O3	0.28657 (6)	0.11763 (6)	0.31043 (15)	0.0336 (3)	
O1S	0.48109 (6)	0.19293 (6)	0.47507 (13)	0.0232 (3)	
O2S	0.52889 (7)	0.10453 (6)	0.27601 (15)	0.0351 (4)	
H2S	0.5524	0.1035	0.3436	0.042*	
C1	0.36431 (9)	0.13635 (8)	0.1537 (2)	0.0264 (4)	
C2	0.38418 (10)	0.14135 (10)	0.0123 (2)	0.0326 (5)	
C3	0.34410 (11)	0.12800 (11)	−0.0989 (2)	0.0439 (6)	
H3A	0.3580	0.1314	−0.1911	0.053*	
C4	0.28728 (11)	0.11077 (11)	−0.0753 (2)	0.0471 (6)	
H4A	0.2614	0.1016	−0.1502	0.057*	
C5	0.26610 (10)	0.10643 (10)	0.0628 (3)	0.0390 (6)	
H5A	0.2258	0.0944	0.0795	0.047*	
C6	0.30315 (9)	0.11932 (9)	0.1725 (2)	0.0294 (5)	
C7	0.44257 (10)	0.16174 (11)	−0.0244 (2)	0.0386 (6)	
H7A	0.4510	0.1629	−0.1203	0.046*	
C8	0.22877 (10)	0.09432 (13)	0.3404 (3)	0.0530 (7)	
H8A	0.1984	0.1170	0.2889	0.080*	
H8B	0.2209	0.0980	0.4394	0.080*	
H8C	0.2271	0.0519	0.3136	0.080*	
C1S	0.46424 (10)	0.14354 (9)	0.5602 (2)	0.0316 (5)	
H1SA	0.4799	0.1497	0.6535	0.047*	
H1SB	0.4809	0.1064	0.5220	0.047*	
H1SC	0.4204	0.1406	0.5635	0.047*	
C2S	0.51222 (14)	0.04601 (11)	0.2403 (3)	0.0646 (9)	
H2SA	0.5060	0.0223	0.3242	0.097*	
H2SB	0.5441	0.0276	0.1848	0.097*	
H2SC	0.4749	0.0471	0.1869	0.097*	

### Atomic displacement parameters (Å^2^)

**Table:**

	*U*^11^	*U*^22^	*U*^33^	*U*^12^	*U*^13^	*U*^23^
Ni	0.02257 (15)	0.02712 (16)	0.01804 (14)	−0.00098 (11)	0.00057 (10)	−0.00374 (10)
O1	0.0258 (7)	0.0310 (8)	0.0241 (7)	−0.0014 (6)	−0.0011 (6)	−0.0027 (6)
O2	0.0285 (8)	0.0531 (10)	0.0218 (7)	−0.0090 (7)	0.0017 (6)	−0.0082 (7)
O3	0.0251 (7)	0.0362 (8)	0.0394 (8)	−0.0014 (6)	0.0064 (7)	0.0024 (7)
O1S	0.0285 (7)	0.0219 (7)	0.0193 (7)	−0.0004 (6)	0.0021 (5)	0.0019 (5)
O2S	0.0365 (8)	0.0299 (8)	0.0388 (9)	0.0023 (7)	−0.0096 (7)	−0.0111 (7)
C1	0.0269 (10)	0.0214 (10)	0.0309 (11)	−0.0003 (8)	−0.0010 (9)	−0.0038 (8)
C2	0.0297 (11)	0.0402 (12)	0.0281 (11)	−0.0042 (10)	0.0004 (9)	−0.0072 (9)
C3	0.0423 (14)	0.0598 (16)	0.0296 (12)	−0.0085 (12)	−0.0021 (10)	−0.0117 (11)
C4	0.0391 (14)	0.0616 (17)	0.0407 (14)	−0.0098 (12)	−0.0123 (11)	−0.0104 (12)
C5	0.0239 (11)	0.0391 (13)	0.0541 (15)	−0.0040 (9)	−0.0052 (10)	−0.0025 (12)
C6	0.0278 (11)	0.0254 (10)	0.0349 (12)	0.0017 (9)	0.0020 (9)	−0.0007 (9)
C7	0.0370 (13)	0.0560 (15)	0.0228 (11)	−0.0058 (12)	0.0034 (9)	−0.0085 (10)
C8	0.0245 (12)	0.0724 (18)	0.0621 (17)	−0.0014 (12)	0.0080 (12)	0.0126 (14)
C1S	0.0412 (12)	0.0259 (11)	0.0276 (11)	−0.0004 (10)	0.0049 (10)	0.0053 (9)
C2S	0.0657 (19)	0.0344 (15)	0.094 (2)	−0.0018 (13)	−0.0241 (16)	−0.0189 (15)

### Geometric parameters (Å, °)

**Table:**

Ni—O1	2.0020 (14)	C2—C3	1.431 (3)
Ni—O1S^i^	2.0350 (13)	C3—C4	1.342 (3)
Ni—O2	2.0522 (15)	C3—H3A	0.9500
Ni—O1S^ii^	2.0568 (13)	C4—C5	1.424 (3)
Ni—O1S	2.0636 (13)	C4—H4A	0.9500
Ni—O2S	2.0938 (14)	C5—C6	1.377 (3)
O1—C1	1.305 (2)	C5—H5A	0.9500
O2—C7	1.239 (3)	C7—H7A	0.9500
O3—C6	1.389 (2)	C8—H8A	0.9800
O3—C8	1.418 (3)	C8—H8B	0.9800
O1S—C1S	1.425 (2)	C8—H8C	0.9800
O1S—Ni^ii^	2.0350 (13)	C1S—H1SA	0.9800
O1S—Ni^i^	2.0568 (13)	C1S—H1SB	0.9800
O2S—C2S	1.398 (3)	C1S—H1SC	0.9800
O2S—H2S	0.8400	C2S—H2SA	0.9800
C1—C6	1.425 (3)	C2S—H2SB	0.9800
C1—C2	1.445 (3)	C2S—H2SC	0.9800
C2—C7	1.423 (3)		
			
O1—Ni—O1S^i^	172.97 (5)	C4—C3—C2	121.3 (2)
O1—Ni—O2	91.00 (5)	C4—C3—H3A	119.4
O1S^i^—Ni—O2	92.41 (5)	C2—C3—H3A	119.4
O1—Ni—O1S^ii^	91.71 (5)	C3—C4—C5	119.5 (2)
O1S^i^—Ni—O1S^ii^	81.74 (6)	C3—C4—H4A	120.3
O2—Ni—O1S^ii^	97.63 (6)	C5—C4—H4A	120.3
O1—Ni—O1S	93.78 (5)	C6—C5—C4	120.98 (19)
O1S^i^—Ni—O1S	82.85 (5)	C6—C5—H5A	119.5
O2—Ni—O1S	175.22 (6)	C4—C5—H5A	119.5
O1S^ii^—Ni—O1S	82.32 (5)	C5—C6—O3	125.44 (18)
O1—Ni—O2S	97.51 (6)	C5—C6—C1	121.94 (19)
O1S^i^—Ni—O2S	88.72 (5)	O3—C6—C1	112.62 (17)
O2—Ni—O2S	88.67 (6)	O2—C7—C2	128.4 (2)
O1S^ii^—Ni—O2S	168.76 (5)	O2—C7—H7A	115.8
O1S—Ni—O2S	90.63 (5)	C2—C7—H7A	115.8
C1—O1—Ni	125.87 (12)	O3—C8—H8A	109.5
C7—O2—Ni	125.47 (14)	O3—C8—H8B	109.5
C6—O3—C8	116.67 (18)	H8A—C8—H8B	109.5
C1S—O1S—Ni^ii^	119.50 (11)	O3—C8—H8C	109.5
C1S—O1S—Ni^i^	120.73 (12)	H8A—C8—H8C	109.5
Ni^ii^—O1S—Ni^i^	97.91 (6)	H8B—C8—H8C	109.5
C1S—O1S—Ni	119.72 (12)	O1S—C1S—H1SA	109.5
Ni^ii^—O1S—Ni	97.19 (5)	O1S—C1S—H1SB	109.5
Ni^i^—O1S—Ni	96.50 (5)	H1SA—C1S—H1SB	109.5
C2S—O2S—Ni	129.19 (15)	O1S—C1S—H1SC	109.5
C2S—O2S—H2S	109.5	H1SA—C1S—H1SC	109.5
Ni—O2S—H2S	113.6	H1SB—C1S—H1SC	109.5
O1—C1—C6	118.61 (18)	O2S—C2S—H2SA	109.5
O1—C1—C2	125.64 (18)	O2S—C2S—H2SB	109.5
C6—C1—C2	115.75 (18)	H2SA—C2S—H2SB	109.5
C7—C2—C3	116.59 (19)	O2S—C2S—H2SC	109.5
C7—C2—C1	122.81 (19)	H2SA—C2S—H2SC	109.5
C3—C2—C1	120.55 (19)	H2SB—C2S—H2SC	109.5
			
O1S^i^—Ni—O1—C1	109.0 (4)	O1S^i^—Ni—O2S—C2S	−161.5 (2)
O2—Ni—O1—C1	−10.03 (15)	O2—Ni—O2S—C2S	−69.1 (2)
O1S^ii^—Ni—O1—C1	87.64 (15)	O1S^ii^—Ni—O2S—C2S	166.6 (3)
O1S—Ni—O1—C1	170.05 (15)	O1S—Ni—O2S—C2S	115.7 (2)
O2S—Ni—O1—C1	−98.82 (15)	Ni—O1—C1—C6	−168.91 (13)
O1—Ni—O2—C7	6.22 (19)	Ni—O1—C1—C2	10.5 (3)
O1S^i^—Ni—O2—C7	−167.63 (19)	O1—C1—C2—C7	−3.7 (3)
O1S^ii^—Ni—O2—C7	−85.64 (19)	C6—C1—C2—C7	175.7 (2)
O1S—Ni—O2—C7	−174.7 (6)	O1—C1—C2—C3	178.9 (2)
O2S—Ni—O2—C7	103.71 (19)	C6—C1—C2—C3	−1.7 (3)
O1—Ni—O1S—C1S	46.98 (14)	C7—C2—C3—C4	−177.3 (2)
O1S^i^—Ni—O1S—C1S	−139.22 (15)	C1—C2—C3—C4	0.2 (4)
O2—Ni—O1S—C1S	−132.1 (7)	C2—C3—C4—C5	0.8 (4)
O1S^ii^—Ni—O1S—C1S	138.20 (15)	C3—C4—C5—C6	−0.3 (3)
O2S—Ni—O1S—C1S	−50.59 (14)	C4—C5—C6—O3	178.63 (19)
O1—Ni—O1S—Ni^ii^	−83.07 (6)	C4—C5—C6—C1	−1.3 (3)
O1S^i^—Ni—O1S—Ni^ii^	90.730 (9)	C8—O3—C6—C5	7.4 (3)
O2—Ni—O1S—Ni^ii^	97.9 (7)	C8—O3—C6—C1	−172.74 (18)
O1S^ii^—Ni—O1S—Ni^ii^	8.15 (6)	O1—C1—C6—C5	−178.32 (19)
O2S—Ni—O1S—Ni^ii^	179.37 (6)	C2—C1—C6—C5	2.2 (3)
O1—Ni—O1S—Ni^i^	178.07 (5)	O1—C1—C6—O3	1.8 (2)
O1S^i^—Ni—O1S—Ni^i^	−8.13 (6)	C2—C1—C6—O3	−177.69 (17)
O2—Ni—O1S—Ni^i^	−1.0 (7)	Ni—O2—C7—C2	−2.4 (4)
O1S^ii^—Ni—O1S—Ni^i^	−90.708 (8)	C3—C2—C7—O2	176.9 (2)
O2S—Ni—O1S—Ni^i^	80.51 (6)	C1—C2—C7—O2	−0.6 (4)
O1—Ni—O2S—C2S	21.8 (2)		

Symmetry codes: (i) −*y*+3/4, *x*−1/4, −*z*+3/4; (ii) *y*+1/4, −*x*+3/4, −*z*+3/4.

### Hydrogen-bond geometry (Å, °)

**Table:**

*D*—H···*A*	*D*—H	H···*A*	*D*···*A*	*D*—H···*A*
O2S—H2S···O1^i^	0.84	2.05	2.8062 (19)	150
O2S—H2S···O3^i^	0.84	2.50	3.181 (2)	139
C5—H5A···O3^iii^	0.95	2.45	3.360 (3)	159
C1S—H1SC···O2S^ii^	0.98	2.47	3.106 (3)	122

Symmetry codes: (i) −*y*+3/4, *x*−1/4, −*z*+3/4; (iii) −*y*+1/4, *x*−1/4, *z*−1/4; (ii) *y*+1/4, −*x*+3/4, −*z*+3/4.
